# Spatial Abundance and Distribution of Potential Microbes and Functional Genes Associated with Anaerobic Mineralization of Pentachlorophenol in a Cylindrical Reactor

**DOI:** 10.1038/srep19015

**Published:** 2016-01-11

**Authors:** Zhi-Ling Li, Jun Nan, Cong Huang, Bin Liang, Wen-Zong Liu, Hao-Yi Cheng, Chunfang Zhang, Dongdong Zhang, Deyong Kong, Kyoko Kanamaru, Tetsuo Kobayashi, Ai-Jie Wang, Arata Katayama

**Affiliations:** 1 State Key Laboratory of Urban Water Resources and Environment, Harbin Institute of Technology, Harbin 150090 China; 2 Institute of Materials and Systems for Sustainability, Nagoya University, Chikusa, Nagoya 464-8603 Japan; 3 Key Laboratory of Environmental Biotechnology, Research Center for Eco-Environmental Sciences, Chinese Academy of Sciences, Beijing 100085, P.R. China; 4 Department of Biological Mechanisms and Functions, Graduate School of Bioagricultural Sciences, Nagoya University, Chikusa, Nagoya 464–8601 Japan; 5 Department of Civil Engineering, Graduate School of Engineering, Nagoya University, Chikusa, Nagoya 464–8603 Japan

## Abstract

Functional interplays of microbial activity, genetic diversity and contaminant transformation are poorly understood in reactors for mineralizing halogenated aromatics anaerobically. Here, we investigated abundance and distribution of potential microbes and functional genes associated with pentachlorophenol (PCP) anaerobic mineralization in a continuous-flow cylindrical reactor (15 cm in length). PCP dechlorination and the metabolite (phenol) were observed at segments 0–8 cm from inlet, where key microbes, including potential reductive dechlorinators (*Dehalobacter*, *Sulfurospirillum*, *Desulfitobacterium* and *Desulfovibrio* spp.) and phenol degraders (*Cryptanaerobacter* and *Syntrophus* spp.), as well as putative functional genes, including putative chlorophenol reductive dehalogenase (*cprA*) and benzoyl-CoA reductase (*bamB*), were highly enriched simultaneously. Five types of putative *cprA*s, three types of putative *bamB*s and seven types of putative nitrogenase reductase (*nifHs*) were determined, with their copy numbers decreased gradually from inlet to outlet. Distribution of chemicals, bacteria and putative genes confirmed PCP dechlorination and phenol degradation accomplished in segments 0–5 cm and 0–8 cm, respectively, contributing to a high PCP mineralization rate of 3.86 μM d^−1^. Through long-term incubation, dechlorination, phenol degradation and nitrogen fixation bacteria coexisted and functioned simultaneously near inlet (0–8 cm), verified the feasibility of anaerobic mineralization of halogenated aromatics in the compact reactor containing multiple functional microbes.

Halogenated aromatic compounds (HACs) are noteworthy for their characteristics of high toxicity, persistence and bioaccumulation. They have led to the serious contamination upon the extensive applications^1^. Twenty-three types of halogenated benzenes or phenols have been recorded as the priority pollutants by the US Environmental Protection Agency, which occupied 17% of the total list^2^. Seeking for the efficient remediation strategies became an urgent and tough assignment to completely remove the toxiticity of HACs in worldwide.

The use of mciroorganisms in detoxification of HACs has addressed the increasing attention as a promising and cost-effective bioremedaition approach^3^. Several aerobic isolates, such as *Pseudomonas* or *Burkholderia* spp., could mineralize HACs through oxidative or hydrolytic dechlorination and oxygenolysis; meanwhile, the related enzymatic genes were also well elucidated^3^,^4^. The biodegradation of HACs, has been extensively investigated in both bioreactors and batch cultures^5^,^6^. With pentachlorophenol (PCP) as an example, under aerobic condition, PCP could be oxidized to chlorohydroquinone accompanied with the removal of chloride and aromatic ring cleavage, and then completely mineralized to CO_2_^7^. Under anaerobic conditions, reductive dehalogenation by replacing the halogen with hydrogen atom played a vital role for converting HACs to the less/none halogenated ones^8^,^9^. Several isolates have been reported that possessed the reductive dehalogenation activities, like *Desulfitobacterium* spp., *Dehalobacter* spp., *Desulfovibrio* spp., *Dehalococcoides* spp., and *Dehalobacter* spp.^10^. However, after reductive dehalogenation, the less or none halogenated metabolites still carry aromatic rings and toxicity. Anaerobic oxidative degradation of these metabolites to CO_2_ is another obligatory bioprocess to completely remove the toxicity of HACs anaerobically^11^.

Hence, artificial combination of two bioprocesses including reductive dehalogenation and anaerobic oxidative degradation is an essential means to realize the complete bio-mineralization of HACs anaerobically. Previously, some anaerobic mineralization systems have been constructed by synthesizing consortia for reductive dehalogenation and oxidative degradation, which acquired the favorable and efficient mineralization activities^11^,^12^,^13^,^14^. However, the two bioprocesses, reductive dehalogenation and oxidative degradation, went through the distinct metabolic pathways and nutrient requirements, and their working mechanism during HACs transformation were little known. So identification of the interplays of responsible bacteria, functional genes and organic chemicals, are vital to better understand the competitive or cooperative interrelations of functional bacteria. The study would give suggestions for the adaptability and regulation strategy for complete mineralization of HACs by alignment of bacteria and nutrients with different functions in contaminated sites or bioreactors.

In this study, a long-term maintained cylindrical reactor for pentachlorophenol (PCP) mineralization utilizing lactate as the sole external nutrient was disassembled. The spatial ecophysiological characterizations of microbes and genes at different cylindrical segments were carried out. The abundance and distribution of organic compounds (PCP, lactate and their corresponding organic metabolites), involved bacteria (reductive dechlorinators and oxidative degraders) and functional genes (chlorophenol reductive dehalogenase cprA, phenol anaerobic degradation benzoyl-CoA reductase/ring-cleavage hydrolase genes *bamB*, *bzdN*, *bcrC* and *bamA*, and nitrogenase gene *nifH*) inside the cylindrical reactor, were extensively investigated. The objects of this study were to (i) reveal the distribution and diversity of the functional bacteria and genes associated with the mineralization of HACs in the constructed reactor and (ii) give understanding on the occurrence and functional mechanisms under the roles of potential bacteria and genes during HACs-mineralization process.

## Results

### Spatial distributions of PCP, lactate and their organic metabolites

**Figure 1:**
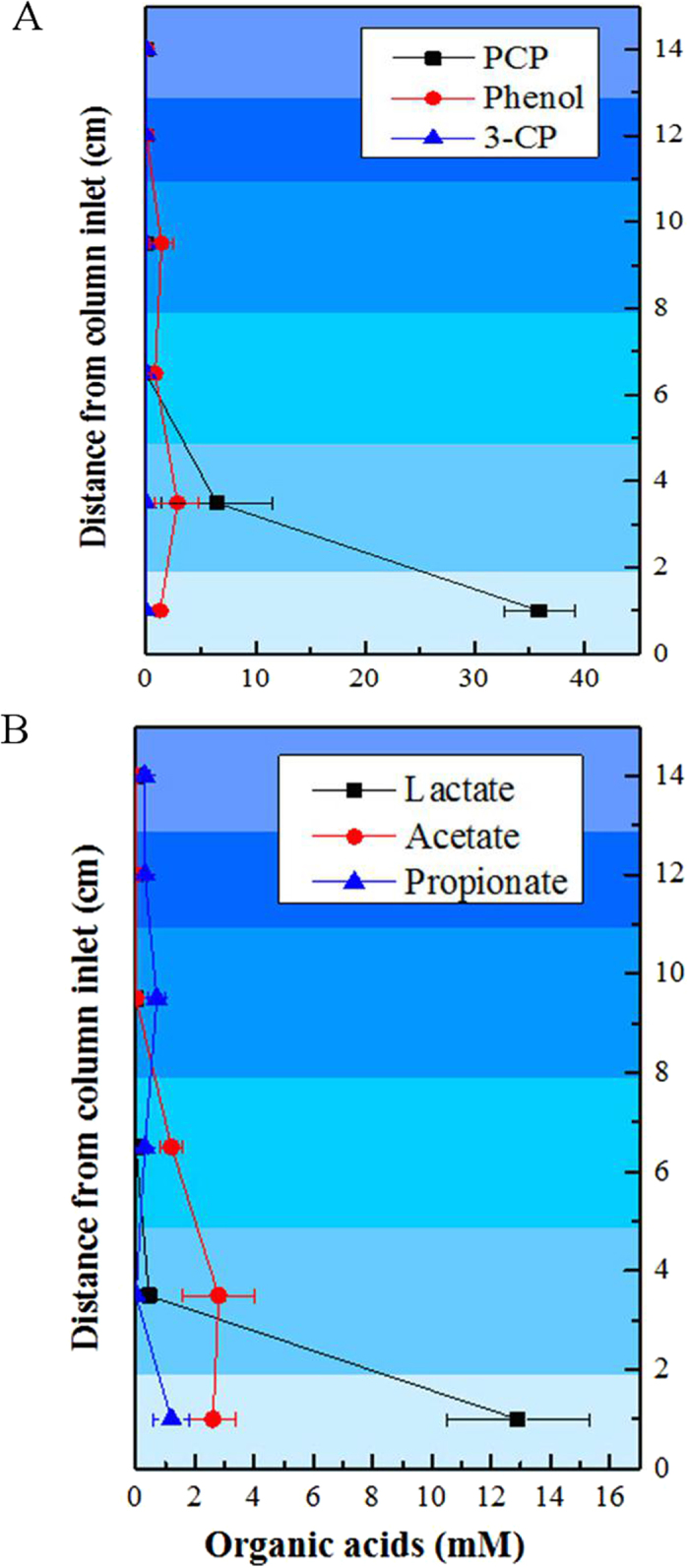
Spatial distributions of PCP, lactate and their corresponding organic metabolites in segments of 0–2 cm, 2–5 cm, 5–8 cm, 8–11 cm, 11–13 cm and 13–15 cm, respectively, along with the cylindrical reactor. (**A**) Concentration changes of PCP, 3-chlorophenol and phenol; (**B**) Concentration changes of lactate, acetate and propionate.

Concentration distributions of PCP, lactate and their organic metabolites were shown in Fig. 1. PCP concentration decreased quickly and became below the detection limit in segment 5–8 cm from the inlet, and while, phenol was detected at a trace concentration less than 5 μM between 0–8 cm (Fig. 1A), indicating the occurrence of reductive dechlorination between 0–5 cm. Besides phenol, none of the other possible metabolites, like 3-chlorophenol (3-CP), 3,5-dichlorophenol or 2,3,5-trichlorophenol, was detectable in the whole reactor segments (Fig. 1A; data not shown). Above segment 8 cm, no phenol was detected, indicating phenol complete degradation accomplished between 0–8 cm. Concentration of lactate decreased quickly between 0–8 cm, where its metabolites, acetate and propionate, were frequently detetced in the range of 0–3 μM and 0–1.8 μM, respectively. Acetate and propionate disappeared in the segment 8–11 cm and 13–15 cm, respectively (Fig. 1B).

### Spatial distribution of potential reductive dehalogenators and phenol anaerobic degraders

**Figure 2:**
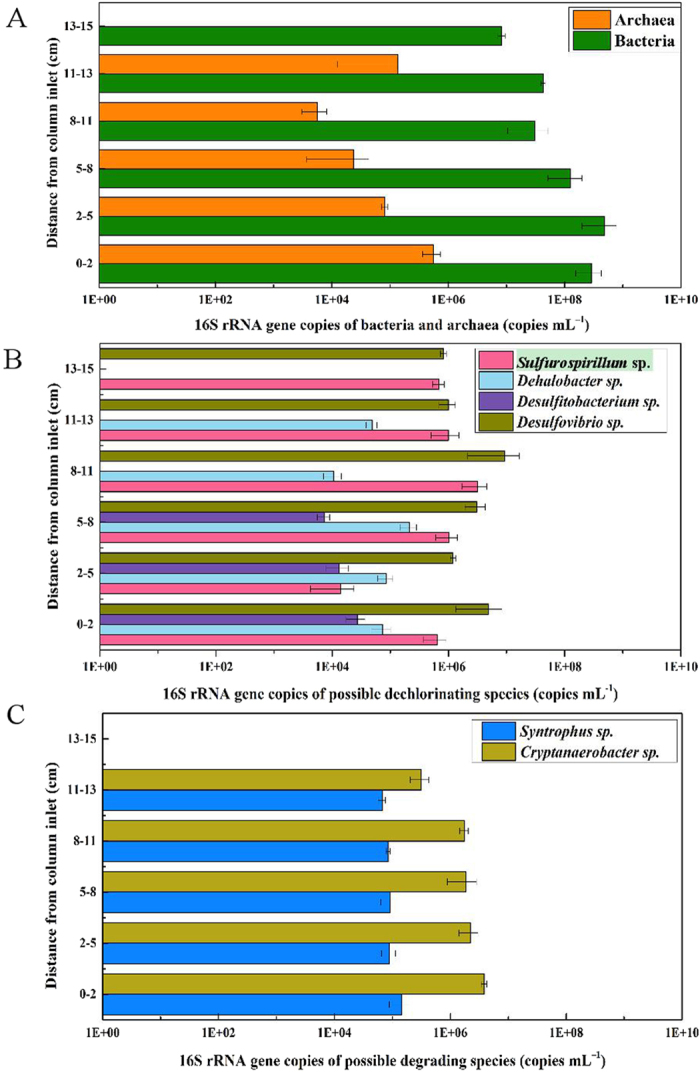
Spatial distributions of Bacteria, Archaea, potential reductive dehalogenators and phenol oxidative degraders in segments of 0–2 cm, 2–5 cm, 5–8 cm, 8–11 cm, 11–13 cm and 13–15 cm, respectively. (**A**) 16S rRNA genes copies of Bacteria and Archaea; (B) 16S rRNA genes copies of possible reductive dehalogenase; (**C**) 16S rRNA genes copies of possible phenol degraders.

Figure 2 represented the distribution of 16S rRNA genes of the genera of potential dehalogenators, *Desulfovibrio*, *Sulfurospirillum*, *Dehalobacter* and *Desulfitobacterium* spp., as well as the potential fermentative or syntrophic degraders of phenol/3-CP, *Cryptanaerobacter* and *Syntrophus* spp. along with the cylindrical reactor. The highest bacterial density appeared in the inlet segment (0–2 cm) (about 10^8^ copies mL^–1^) and dropped to about 10^6^ copies mL^–1^ near the outlet segment (13–15 cm) (Fig. 2A). Archaea was detected with the density ranged from 10^3^ to 10^6^ copies mL^–1^, except for the outlet segment (13–15 cm).

*Desulfovibrio* and *Sulfurospirillum* spp., known as the facultative dechlorinators^15^,^16^, were evenly distributed among the whole reactor, and occupied 0.2–30.3% and 0.2–8.3% of the copy number of Bacteria, respectively. Both of their highest densities were appeared at the segment 8–11 cm, and while no obvious density decrease was observed where PCP was disappeared above 5 cm of segment (Fig. 1A). In contrast, *Dehalobacter* and *Desulfitobacterium* spp., the two dehalogenators^17^,^18^, occupied the much lower properties of bacterial composition (0.02–0.2% and 0.002–0.01% of the copy number of Bacteria, respectively). However, their density profiles were consistent with the distribution of PCP (Fig. 1A). Both of them showed a great decrease of wider than 10 times in the segments from 8 cm, and were below the detection limit above 13 cm and 8 cm, respectively (Fig. 1A). Correlation analysis showed *Dehalobacter* and *Desulfitobacterium* spp. were positively correlated with the concentration of PCP, with the pearson correlation constant *r* of 0.22 and 0.94, respectively. However, no obvious correlation was observed for *Desulfovibrio* and *Sulfurospirillum* spp. It is inferred *Dehalobacter* and *Desulfitobacterium* spp. were involved in reductive dechlorination process, and while, *Desulfovibrio* and *Sulfurospirillum* spp., were probably participated in the other bioprocesses, such as fermentation or nitrogen fixation^17^,^18^.

Copy numbers of the two possible phenol degraders, *Cryptanaerbacter* and *Syntrophus* spp., were gradually decreased from the inlet to outlet, and occupied 0.1–0.3% and 0.4–5.4% of the copy number of Bacteria, respectively (Fig. 2C). None of them was detected in the outlet segment of 13–15 cm. Changes in numbers of *Cryptanaerbacter* and *Syntrophus* spp. were consistent with the distribution of Archaea, indicating their latent syntrophic relationships. Also, gene copy numbers of both *Cryptanaerbacter* and *Syntrophus* spp. were significantly correlated with the determined concentration of phenol (*r* = 0.63 and 0.51, respectively). So it is inferred *Cryptanaerbacter* and *Syntrophus* spp. were in associated with phenol syntrophic degradation.

### PCR amplification and phylogenetic analysis of *cprA* genes

The presence of *cprA* genes was examined using the extracted DNA from the inlet segment 0–2 cm with the designed primers. About 400–500 bp lengths of products were successfully amplified with the designed primer pairs, except for a primer pair of f5-m3-g2 and r3-m3-g2 (SI Table S4). Through clone library analysis, genes that possessing the following two traits were judged as the putative *cprA* reductive dehalogenases: that is (i) possessed two-conserved iron-sulfur cluster binding motifs and (ii) shared the highest similarity with the reported *cprA* genes from the BLAST program of GenBank nucleotide sequence database compared other functional genes. Over 90% of amplified genes were regarded as the putative *cprA* genes, indicating the validity of the designed primers used. No positive amplicon was observed with the specific primer sets for *cprA3*, *cprA4* and *cprA5* (SI Table S4) identified in *Desulfitobacterium hafniense* PCP-1^17^, suggesting the lack of these genes in the reactor.

**Figure 3:**
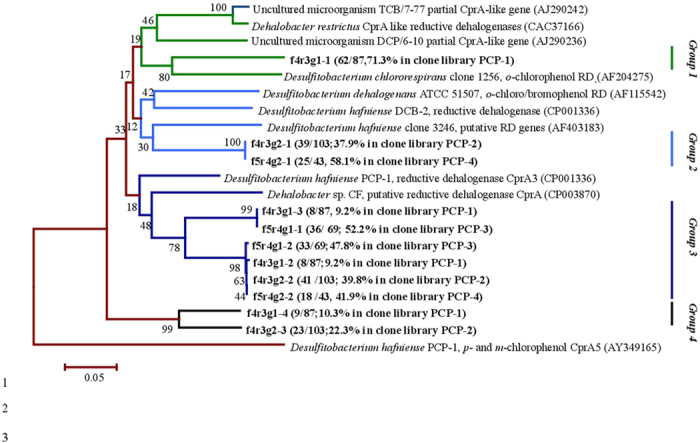
Phylogenetic tree of the putative cprA genes determined from the four groups of clone libraries PCP-1 to PCP-4 in the inlet segment (0–2 cm) of the reactor (SI Table S4) and their closest relatives, reductive dehalogenase cprA genes. The branch in bold represents the gene types, with the appearance frequency as shown in brackets. The numbers at the nodes indicate the percentages of times that nodes appeared in 1000 bootstrap analyses. The scale bar represents an estimated difference of 10% in nucleotide sequence positions.

Four clone libraries, PCP-1, PCP-2, PCP-3 and PCP-4, were constructed with the gene fragments amplified by primer sets of f4-m3-g1 and r3-m3-g1, f4-m3-g2 and r3-m3-g2, f5-m3-g1 and r4-m1, and f5-m3-g2 and r4-m1, respectively (SI Table S4). Representative genes were majorly classified into five types (Fig. 3) and their highest similarities with the reported *cprA* genes were shown in SI Table S5. Gene f4r3g1-1 was the most abundant in PCP-1 and possessing the highest similarity with the *o*-position chlorophenol reductase in *Desulfitobacterium chlororespirans* clone 1256 (75.7%) harboring two iron-sulfur clusters. Genes of f4r3g2-1 and f5r4g2-1 in clone libraries of PCP-2 and PCP-4, respectively, were of 100% similarity with each other and the most associated with the determined *o*-chloro/bromophenol *cprA* of *Desulfitobacterium dehalogenans*. F4r3g1-3 and f5r4g1-1 in clone libraries of PCP-1 and PCP-3 possessed less than 80% of similarities with *o*-chloro/bromophenol *cprA* of *D. dehalogenans*, *cprA3* of *Desulfitobacterium hafniense* PCP-1, and putative *cprA* genes of *Dehalobacter* sp. CF, respectively. F5r4g1-2, f4r3g1-2, f4r3g2-2 and f5r4g2-2 were over 98% similar with each other and the most related to *cprA* genes specific for *o*-chloro/bromophenol in *D. dehalogenans*. Genes in group four, f4r3g1-4 and f4r3g2-3, harbored over 99% of the similarity with each other, and both of them possessed the highest similarity with *cprA* genes determined in *Dehalobacter restrictus*.

### PCR amplification and phylogenetic analysis of *bcr* and *bamA* genes

DNAs extracted on the inlet segment (0–2 cm) and outlet segment (13–15 cm) were examined for the genes associated with the central intermediate of benzoyl-coenzyme A (CoA) pathway in phenol degradation, including the benzoyl-coenzyme A reductases (BCR) of *bamB*, *bzdN* and *bcrC* and the benzoyl-CoA ring-cleaving hydrolase of *bamA*^19^. Positive amplicons with the correct size were detected for *bamB* gene at 0–2 cm of the segment, but no amplicon was found for *bzdN*, *bcrC*, or *bamA* genes in both the inlet and outlet segments (SI Table S6), indicating the lack of these genes there. The primer set for *bamB* genes was rather conserved^19^ and over 95% of the picked colonies shared the highest similarities with the identified *bamB* genes through clone library analysis. As *bamB* codes for BCR subunit in obligate anaerobes, and while *bzdN* or *bcrC* targets for the facultative anaerobes^19^,^20^, the results further confirmed that phenol degradation was carried out through the obligate anaerobes^13^.

**Figure 4:**
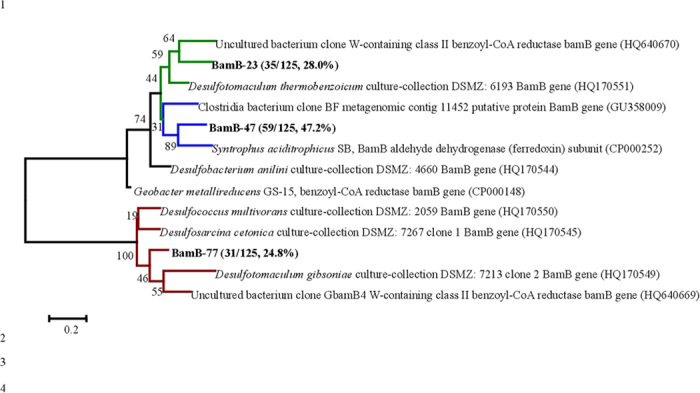
Phylogenetic tree of the benzoyl-CoA reductase putative *bamB* genes determined in the inlet segment (0–2 cm) of reactor and their closest relatives of *bamB* genes reported. The branch in bold represents the gene types, with the appearance frequency as shown in brackets.

Clone library showed the amplified putative *bamB* genes were classified into mainly three types, BamB-23, BamB-47 and BmaB-77, with their closely related *bamB* genes as shown in phylogenetic tree (Fig. 4). Among them, BamB-47 was the most abundant (47.2%) and possessed 77% of similarity with *bamB* aldehyde dehydrogenase of *Syntrophus aciditrophicus* SB. BamB-23 and BamB-77 were closely related to *bamB* genes of *Desulfotomaculum thermobenzoicum* (75% of similarity) and *Desulfotomaculum gibsoniae* (72% of similarity), respectively. The determined putative *bamB* genes were mostly associated with the ones identified in either *Syntrophus* sp. or *Desulfotomaculum* sp. (Fig. 4), further revealed the syntrophic or fermentative features during phenol anaerobic degradation^13^.

### PCR amplification and phylogenetic analysis of *nifH* genes

**Figure 5:**
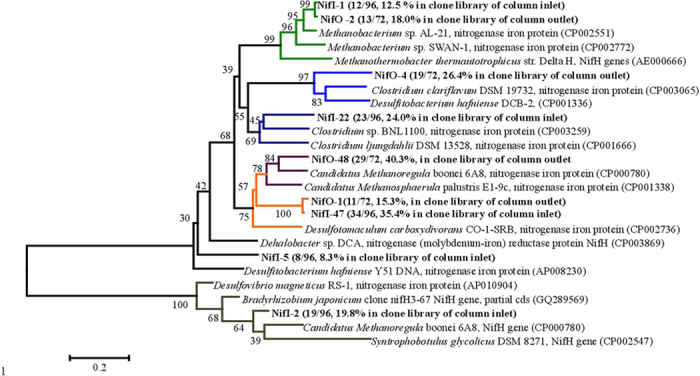
Phylogenetic tree of the nitrogenase putative *nifH* genes determined in the inlet segment (0–2 cm) (clone library NifI) and outlet segment (12–15 cm) (clone library NifO) of the reactor (SI Table S6), as well as their closest related nitrogenase genes.

DNAs on both the inlet (0–2 cm) and outlet (13–15 cm) segments were examined for the presence of highly conserved *nifH* genes encoding iron protein^21^,^22^,^23^. Positive amplicons were obtained in both the inlet and outlet segments (SI Table S6) and over 95% of the picked colonies shared the highest similarities with the identified *nifH* genes. Clone library analysis showed five types (NifI-1, 2, 5, 22 and 47) of putative *nifH* genes in the inlet segment, and four types (NifO-1, 2, 4 and 48) of putative *nifH* genes in the outlet segment, respectively (Fig. 5). Among of them, NifI-47, identical with NifO-1, was the most abundant in the inlet segment (35.4%) and possessed 76% of similarity with nitrogenase determined in *Desulfotomaculum carboxydivorans* strain CO-1-SRB, a benzoate degrader under sulfate reducing environment. The gene NifO-48 was the most abundant in the outlet segment (40.3%) and possessed 83% of similarity with nitrogenase determined in *Methanoregula boonei* strain 6A8. NifI-1 and NifO-2 were of 99% identical in sequence each other and closely related to the nitrogenase determined in *Methanobacterium lacus* strain (91% of similarity). The gene *NifH*-I-2 had the highest identity in DNA sequence with *nifH* genes in *Bradyrhizobium japonicum* clone (75% of similarity), a facultative nitrogen fixation bacterium. The genes, *nifH*-I-5 and *nifH*-I-22 possessed 78% and 75% similarities with genes in *Dehalobacter* sp. DCA and fermentation bacteria *Clostridium* sp. BNL1100, respectively. The gene *nifH*-O-4 possessed 69% similarity with *nifH* gene determined in *Clostridium clariflavum* DSM 19732.

### Spatial distribution of putative functional genes: *cprA*, *bamB* and *nifH*

**Figure 6:**
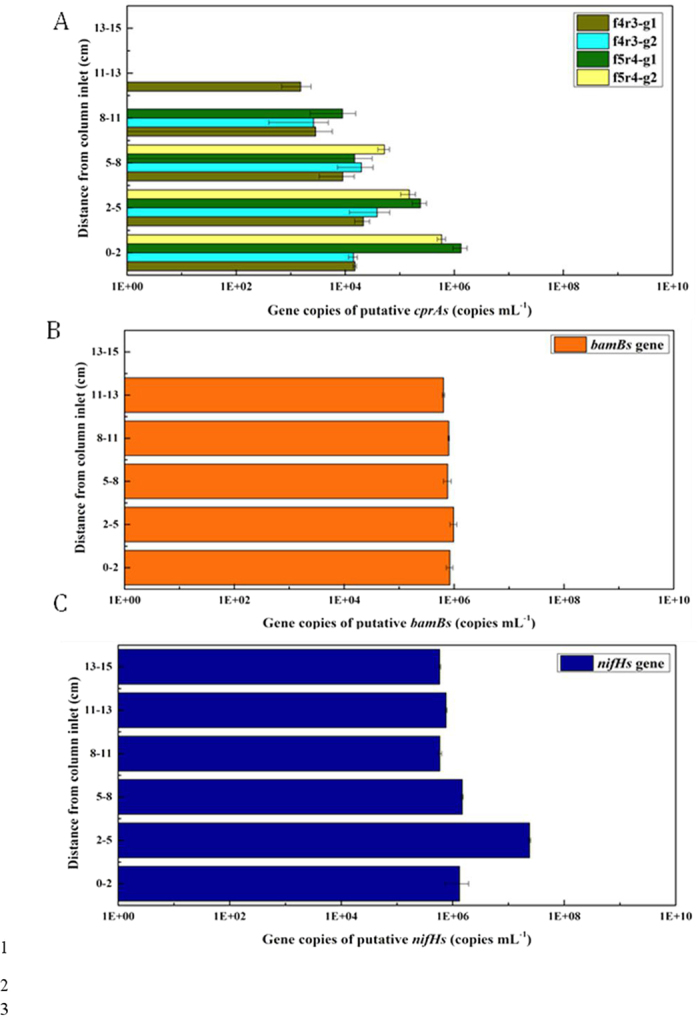
Spatial distributions of putative *cprA*, *nifH* and *bamB* genes in segments of 0–2 cm, 2–5 cm, 5–8 cm, 8–11 cm, 11–13 cm and 13–15 cm, respectively. (**A**) quantitive distribution of *cprA*. F4r3g1, f4r3-g2, f5r4-g1 and f5r4-g1 indicated the amplified products with primer sets of f4-m3-g1/ r3-m3-g1, f4-m3-g2/r3-m3-g2, f5-m3-g1/r4-m1 and f5-m3-g2/r4-m1, respectively; (**B**) quantitive distribution of *bamB*-like genes; (**C**) quantitive distribution of *nifH*-like genes.

Spatial distributions of putative *cprA*, *bamB* and *nifH* genes along with the reactor segments were shown in Fig. 6. Gene copy numbers of putative *cprA* genes amplified from four pairs of primer sets, appeared the highest at the inlet segment 0–2 cm (10^4^–10^6^ copies mL^–1^), and decreased gradually as the distance increase from the inlet segment until below the detection limit at 13–15 cm. Distribution trend of the putative *cprA* genes was coincident with that of distribution of *Dehalobacter* and *Desulfitobacterium* spp. (Fig. 2B). In segments 0–13 cm, genes amplified with f5-m3-g1 and r4-m1 primers were numerically dominant (10^4^–10^6^ copies mL^–1^) except for 5–8 cm, and while, genes amplified by f4-m3-g1 and r3-m3-g1 primers were the most frequently detected, although they possessed a relative low copy number (10^4^–10^6^ copies mL^–1^). Gene obtained by f4-m3-g2 and r3-m3-g2 primers ranged between 10^3^–10^4^ copies mL^–1^ at the segment 0–11 cm and below the detection limit in the segment 11–15 cm. Genes obtained by f5-m3-g2 and r4-m1 primers presented at a rather high copy numbers (10^4^–10^6^ copies mL^–1^), but, they were not detected between 8–15 cm. The correlation analysis showed the sum of determined *cprA* genes were highly correlated with concentration of PCP (*r* = 0.95), which further indicated that *cprA* genes played an important role during PCP reductive dechlorination process.

Putative *bamB* genes were distributed along with the whole segments, except for the segment 13–15 cm (Fig. 6B). Copy numbers of putative *bamB* genes did not show any big fluctuation in the segments 0–13 cm, being consistent with the distributions of the potential phenol syntrophic degraders (Fig. 2C). Copy numbers of putative *bamB* genes were comparable with the sum number of potential phenol syntrophic degraders, *Cryptanaerbacter* or *Syntrophus* spp. The significant correlation of *bamB* genes with concentration of phenol (*r* = 0.74) suggested its involvement in phenol degradation process.

Putative *nifH* genes were distributed widely among the whole reactor, with the highest copy number of appropriate 10^7^ copies mL^–1^ at 2–5 cm and the lowest copy number of 9 × 10^5^ copies mL^–1^ at 13–15 cm, respectively (Fig. 6C). The copy numbers of putative *nifH* genes showed a slight of decrease as the increase in the distance from inlet segment, which suggested the distribution of nitrogen fixing bacteria.

## Discussion

The current study demonstrated the abundance and distribution of representative bacteria, functional genes as well as organic chemicals, which clearly revealed PCP mineralization process accomplished by the cooperative activities of the different bacteria (potential reductive dechlorinators and phenol degraders) and functional genes (putative functional genes of *cprA*, *bamB* and *nifH* genes). The distributions of PCP and phenol (Fig. 1A) suggested PCP dechlorination occurred at the segments 0–5 cm, confirmed by the highest densities of *Dehalobacter*, *Desulfitobacterium* spp. and putative *cprA* genes there (Figs 2B and 6A). Distributions of PCP and phenol revealed the anaerobic degradation of phenol took place at the segments 0–8 cm (Fig. 1A), verified by the distribution trends of both phenol potential degraders and putative *bamB* genes (Figs 2C and 6B). The gradual decrease in density of the potential PCP dechlorinators, phenol degraders and the corresponding genes at the segments longer than 5 cm and 8 cm from the inlet of the reactor (Figs 2B,C and 6A,B) reflected the gradual drop and lose of PCP dechlorination and phenol degradation activities at the middle and outlet segments of the reactor. Meanwhile, the wide distributions, the high copy numbers and diversities of putative *nifH* genes (Figs 5 and 6C) indicated the nitrogen fixation process throughout the reactor.

PCP dechlorination rate was determined as 5.89 μM d^−1^, calculated by multiplying the influent PCP concentration (50 μM) with the flow rate (0.003 mL min^−1^) and dividing by the effective volume where the reductive dechlorination occurred. The effective volume was calculated as 36.7 mL by multiplying the reactor pore volume (110 mL) with the effective length of reductive dechlorination occurred (5 cm) and dividing by the reactor length (15 cm). Therefore, the PCP mineralization in the segments 0–8 cm means 3.86 μM d^−1^ of the PCP mineralization rate, which is much higher than the previous report of 1.96 μM d^−1^ 13, as well as the one achieved in an UASB reactor (0.13 μM d^−1^)^24^ and in a system with two combined columns in series (3.46 μM d^−1^)^11^. Lactate, the sole external nutrient, was completely fermented at the segment 0–5 cm, and the metabolites of acetate and propionate were further decomposed in 0–13 cm (Fig. 1B). As calculated in the same way, lactate mineralization rate was 0.57 mM d^−1^ in this reactor.

It is noticed phenol degradation proceeded simultaneously with PCP reductive dechlorination at the segments 0–5 cm, confirmed by the low phenol concentration (less than 5 μM) and the decrease in concentration of PCP (Fig. 1A). The simultaneous reactions were further verified by the high density of both potential dechlorinating and phenol degrading bacteria, and the corresponding functional genes: putative *cprA* and *bamB* genes (Figs 2C and 6B). Previously, the acclimatization of an anaerobic community capable of mineralizing HACs is considered challenging because the different redox niches required for multiple bioprocesses, especially reductive dechlorination and anaerobic oxidative degradation^11^,^12^,^13^,^14^. This study revealed the coexistence and cooperative work of dechlorinators and anaerobic phenol degraders in an acclimated consortium at 0–8 cm, which satisfied the requirement of simultaneous reduction and oxidation and avoided the internal nutrient competition. Besides, functions of nitrogen-fixing bacteria and lactate-fermenting bacteria^13^ should be mentioned (Figs 1B and 6C), which supplied organic nitrogen for microbial growth and electron (H_2_) for reductive dechlorination, respectively, although the distribution of their related populations and/or functional genes were not thoroughly elaborated.

The correlation analysis suggested *Desulfitobacterium* and *Dehalobacter* spp. were involved in reductive dechlorinating process, and while, *Syntrophus* and *Desulfotomaculum* spp. were in associated with phenol degradation process (Figs 1 and 2). The functional bacterial composition was different from the previous constructed HAC anaerobic mineralization systems. Li et al.^14^ reported the anaerobic mineralization of tribromophenol, through fermentation, reductive debromination and oxidative degradation with *Clostridium* strain Ma13, *Dehalobacter* sp. and *Desulfatiglans* strain DS, respectively. A recent study reported anaerobic mineralization of the wide types of HACs in a long-term maintained anaerobic consortium, and the involved potential dehalogenators were *Dehalobacterium* and *Sulfurospirillum* spp., and while, oxidative degraders were *Geobacter* spp.^25^ This indicated the diverse bacterial community structure for HAC mineralization system. However, the bacterial communities were analyzed by setting up the 16S rRNA clone library, which could only provide the restrictive community information. The recent developed high-throughput sequencing technics with the high taxonomic resolution are warranted to give the comprehensive evaluation of structure and diversity of functional bacteria and genes.

Through clone libraries, five types of *cprA*-like genes, three types of *bamB*-like genes and seven types of *nifH*-like genes were identified in the cylindrical reactor. The applied primers is based on the recent reports or synthesized according to the recently identified genes. So far, studies on identification and characterization of *cprA* and *bamB* genes were just on the beginning, and the identified functional genes were still in limited genomes, i.e. *cprA* genes from the limited dechlorinating bacteria (*Desulfitobacterium* sp., *Dehalobacter* sp.) and *bamB* genes from the obligate anaerobes for aromatic-compounds degradation (*Geobacter* sp., *Syntrophus* sp.)^17^,^18^,^19^,^20^. There is the possibility that other types of functional genes might be present with the more diverse molecular. In fact, various type of reductive dehalogenase homologous have been recently reported existed in single isolates^5^,^26^, reflected that the identified genes with pairs of synthesized primers could not cover all the possible functional dehalogenases, *bamB* or *nifH* genes. Further study is required on the identification, characterization and genetic expression analysis of those functional genes, to extend the understanding of gene functioning mechanism in molecular aspect.

It should be mentioned that the determined putative *nifH* genes were originated from not only nitrogen fixation bacteria but also some other bacteria holding the *nifH* motifs, such as *Dehalobacter* sp., *Methanoregula boonei* and *Bradyrhizobium* sp. (Fig. 5). Nitrogen fixation activity has been reported by some reductive dechlorinators, aromatic-ring degraders or fermenters, and while, their activities were enhanced through nitrogen fixation process^23^,^27^. So there is the possbility that the bacteria in charge of PCP mineralization also participated in nitrogen fixation process, and simutanously supplied organic nitrogen for microbial growth. Compared with the inlet segment (0–2 cm), the outlet segment (13–15 cm) lacked putative *nifH* genes which originated from the potential dechlorinators and aromatic-ring degraders, caused by the lack of the related bacteria there (Fig. 2B).

Collectively, the study gave, for the first time, a clear image on the spatial distribution and diversity of potential reductive dechlorinators, anaerobic oxidative degraders and related functional genes (*cprA*, *bamB* and *nifH*), and represented a sustaining anaerobic bacterial community functioned cooperatively and sequentially to achieve PCP mineralization at the segment 0–8 cm from the column inlet. The study gave suggestions of designing a compact anaerobic HACs mineralization system by applying the multiple functional microbes in porous medium.

## Materials and Methods

### Preparation of the cylindrical reactor and the methods of sample storage

The cylindrical reactor used in this study was described previously^13^. Briefly, a glass beads-packed anaerobic cylindrical reactor (15 cm long) was provided by the inoculation of a consortium reductively dechlorinating PCP and introduction of solutions containing lactate (20 mM) and PCP (50 μM) under continuous up-flowing conditions. The cylindrical reactor performed the dechlorination of PCP to 3-CP and phenol with H_2_ as electron donor generated by lactate fermentation, and then, 3-CP and phenol were further degraded without externally added electron acceptor after extending the hydraulic retention time. Nitrogen required for the microbial growth was supplied by microbial N_2_-fixation process. A 16S rRNA gene library analysis showed that the inlet segment harbored the potential dechlorinators, *Dehalobacter*, *Desulfitobacterium*, *Sulfurospirillum* and *Desulfovibrio* spp., and the phenol/3-CP fermentative or syntrophic degraders, *Cryptanaerobacter* and *Syntrophus*. Some possible nitrogen-fixers were detected in both the inlet and outlet segments.

After continuous running for 1.5 years with HRT of 25.3 days, the cylindrical reactor (15-cm long) was unloaded, crossed-cut into six segments under anoxic condition in a glove box (90% N_2_, 10% H_2_ atmosphere) and stored in −80 °C. Samples were collected as the following intervals from the bottom to the top of cylindrical reactor: 0–2 cm, 2–5 cm, 5–8 cm, 8–11 cm, 11–13 cm and 13–15 cm. The mixture of solid (soil and glass beads) and liquids of each segment was sampled with an autoclaved spatula. The samples were placed in 10 mL polypropylene tubes and stored at −30 °C before the chemical analysis and DNA extraction. The framework of the reactor and the analytical details after reactor decomposition was listed in SI Figure S1.

### Chemical analysis

Initially, glass beads were gently removed from the frozen samples. PCP and the metabolites were determined by GC-MS (Shimadzu, Kyoto, Japan) equipped with the capillary column (DB-5MS, 30 m of length, 0.25 mm of inner diameter, J & W Scientific, Folsom, CA). Organic acids (lactate, acetate and propionate) were determined using a high-performance liquid chromatograph (CTO-10A; Shimadzu, Kyoto, Japan) equipped with an ultraviolet detector (UV 280 nm) and a Puresil C18 column (4.6 mm inner diameter, 250 mm length; Waters, Milford, MA, USA). The detailed method for measuring PCP, lactate and their metabolites were described in supplementary information (SI).

### DNA extraction and the primer design

Initially, glass beads were gently removed from the frozen samples. DNA extraction from the samples (1.5 mL of the total volume of soil and liquid) was carried out using a DNA extraction kit, Isoplant II, according to the manufacturer’s instruction (Nippon Gene Co., Ltd., Toyama, Japan). Primers for the amplification of chlorophenol reductive dehalogenase *cprA* gene fragments, targeting for the binding motifs of the two iron-sulfur clusters were designed modified by combining the recently released *cprA* gene sequences^18^,^28^,^29^,^30^. The *cprA* gene sequences applied for primers design were listed in SI. Information of the sequences, positions and targeted genes of the designed primers were listed in supplementary information (SI) Table S1. Primer sets specific for gene sequences of *cprA3*, *cprA4*, and *cprA5* in *Desulfitobacterium hafniense* DCB-2^17^ were also utilized as listed in SI Table S1. Primer sets of *bamB*, *bzdN*, *bcrC* and *bamA* were applied for amplification of benzoyl-CoA reductase and ring-cleaving hydrolase involved in the anaerobic phenol degradation, with the detailed information as shown in SI Table S2. *BamB* targeted for active-site subunit of class II benzoyl-coenzyme reductases (BCRs) from obligate anaerobes^19^,^20^. *BzdN* and *bcrC* targeted for subunits of class I BCRs from facultative anaerobes (*bcr* referred to as genes coding for *Thauera* type and *bzd* referred to as genes coding for *Azoarcus* type)^19^. *BamA* targeted for ring-cleaving hydrolase of the benzoyl-CoA degradation genes^20^. The primer set utilized for the amplification of the highly conserved nitrogenase was NifH-f and NifH-r targeting *nifH* gene encoding the iron protein (SI Table S2)^21^,^22^. The amplification of putative *cprA* genes, *nifH* genes and benzoyl-CoA related genes were conducted with DNAs extracted from individual segments.

### PCR, cloning and nucleotide sequencing analysis

PCR reactions for *cprA* genes, *nifH* genes, and benzoyl-CoA related genes were performed with ExTaq DNA polymerase kit (Takara Bio Inc., Shiga, Japan). After PCR amplification and the product purification, clone libraries were set up using the pGem-T easy cloning kit (Promega, Madison, WI, USA). The detailed information for PCR program, the product purification and clone library setting up were described in in detail in SI. The clones were categorized on the basis of their distinct RFLPs. The representative clones with a unique RFLP pattern were sequenced with the BigDye terminatorv 3.1 cycle sequencing kit (Applied Biosystems, Foster, CA, USA) using an ABI 3100DNA sequencer (Applied Biosystems, Foster, CA, USA). The obtained nucleotide sequences were aligned using BioEdit Sequence Alignment Editor (7.0.5.3, Ibis Biosciences, Carlsbad, CA, USA) and classified into one type at a level of sequence similarity of more than 98%. The classification and analysis of nucleotide sequence were described in detail in SI. The nucleotide sequences obtained from clone library have been deposited in DNA Data Bank of Japan nucleotide sequence databases under accession numbers from LC033461 to LC033477.

### Quantitative PCR (*q*PCR) analysis

*Q*PCRs were performed using a Light Cycler system (Roche Diagnostics, Mannheim, Germany) and a Light Cycler Fast-start DNA Master SYBR green I kit (Roche Molecular Biochemicals, Indianapolis, IN), as described previously^13^. Bacteria, Archaea, *Dehalobacter* sp., *Sulfurospirillum* sp., *Desulfitobacterium* sp., *Desulfovibrio* sp., *Cryptanaerobacter* sp., and *Syntrophus* sp. were quantified using specific primer sets targeting for 16s rRNA genes as shown in SI Table S3. The putative *cprA* genes were quantified using the primer sets of f4-m3-g1/r3-m3-g1, f4-m3-g2/r3-m3-g2, f5-m3-g1/r4-m1 and f5-m3-g2/r4-m1, shown in SI Table S1. The putative *nifH*- genes and putative *bamB* genes were also quantified using the primer sets of NifH-f/NifH-r and BamB-f/BamB-r, respectively, as shown in SI Table S2. The detailed information on calibration curves and detection limits were described in detail in SI. Triplicate cultures were examined for each condition.

## Additional Information

**How to cite this article**: Li, Z. *et al*. Spatial Abundance and Distribution of Potential Microbes and Functional Genes Associated with Anaerobic Mineralization of Pentachlorophenol in a Cylindrical Reactor. *Sci. Rep*. **6**, 19015; doi: 10.1038/srep19015 (2016).

## Supplementary Material

Supplementary Information

## References

[b1] Sim W. J., Lee S. H., Lee I. S., Choi S. D. & Oh J. E. Distribution and formation of chlorophenols and bromophenols in marine and river environments. Chemosphere. 77, 552−558 (2009). 19664797 10.1016/j.chemosphere.2009.07.006

[b2] US EPA (US Environmental Protection Agency). Ground water and drinking water. National Primary Drinking Water Standards, 2003. EPA-816-F-03-016. USEPA, Office of Water, Washington, DC, USA.

[b3] Field J. A. & Sierra-Alvarez R. Microbial degradation of chlorinated benzenes. Biodegradation. 19, 463−480 (2008). 17917704 10.1007/s10532-007-9155-1

[b4] Potrawfke T., Timmis K. N. & Wittich R. M. Degradation of 1,2,3,4-tetrachlorobenzene by *Pseudomonas chlororaphis* RW71. Appl. Environ. Microb. 64, 3798−3806 (1998). 10.1128/aem.64.10.3798-3806.1998 PMC106552 9758802

[b5] Fricker A. D., LaRoe S. L., Shea M. E. & Bedard D. L. *Dehalococcoides mccartyi* strain JNA dechlorinates multiple chlorinated phenols including pentachlorophenol and harbors at least 19 reductive dehalogenase homologous genes. Environ. Sci. Technol. 48, 14300−14308 (2015). 25377868 10.1021/es503553f

[b6] Huang L. P. . Mineralization of pentachlorophenol with enhanced degradation and power generation from air cathode microbial fuel cells. Biotechnol. Bioeng. 109, 2211−2221 (2012). 22392229 10.1002/bit.24489

[b7] Kazuhiro K. . Aerobic mineralization of hexachlorobenzene by newly isolated pentachloronitrobenzene-degrading *Nocardioides* sp. strain PD653. Appl. Environ. Microbiol. 75, 4452−4458 (2009). 19429557 10.1128/AEM.02329-08 PMC2704805

[b8] Bhatt P., Kumar Suresh M., Mudliar S. & Chakrabarti T. Biodegradation of chlorinated compounds−a review. Crit. Rev. Environ. Sci. Technol. 37, 165−198 (2007).

[b9] Hollinger C., Wohlfarth G. & Diekert G. Reductive dechlorination in the energy metabolism of anaerobic bacteria. FEMS Microbiol. Rev. 22, 383−398 (1999).

[b10] Li Z. . Involvement of Dehalobacter strains in the anaerobic dechlorination of 2,4,6-trichlorophenol. J Biosci. Bioeng. 116, 602−609 (2015). 23777715 10.1016/j.jbiosc.2013.05.009

[b11] Li Z. L., Yang S. Y., Inoue Y., Yoshida N. & Katayama A. Complete anaerobic mineralization of pentachlorophenol (PCP) under continuous flow conditions by sequential combination of PCP-dechlorinating and phenol-degrading consortia. Biotechnol. Bioeng. 107, 775−785 (2010). 20589845 10.1002/bit.22841

[b12] Yang S., Shibata A., Yoshida N. & Katayama A. Anaerobic mineralization of pentachlorophenol (PCP) by combining PCP-dechlorinating and phenol-degrading cultures. Biotechnol. Bioeng. 102, 81−90 (2009). 18683261 10.1002/bit.22032

[b13] Li Z. L., Inoue Y., Suzuki D., Ye L. & Katayama A. Long-term anaerobic mineralization of pentachlorophenol in a continuous-flow system using only lactate as an external nutrient. Environ. Sci. Technol. 47, 1534−1541 (2013). 23252798 10.1021/es303784f

[b14] Li Z. L. . Anaerobic mineralization of 2,4,6-tribromophenol to CO_2_ by a synthetic microbial community comprising *Clostridium*, *Dehalobacter*, and *Desulfatiglans*. Bioresour. Technol. 176, 225−232 (2015). 25461007 10.1016/j.biortech.2014.10.097

[b15] Drzyzga D. & Gottschal J. C. Tetrachloroethene dehalorespiration and growth of *Desulfitobacterium frappieri* TCE1 in strict dependence on the activity of *Desulfovibrio fructosivorans*. Appl. Environ. Microbiol. 68, 642−649 (2002). 11823202 10.1128/AEM.68.2.642-649.2002 PMC126694

[b16] Renpenning J. . Combined C and Cl isotope effects indicate differences between corrinoids and enzyme (*Sulfurospirillum multivorans PceA*) in reductive dehalogenation of Tetrachloroethene, but not Trichloroethene. Environ. Sci. Technol. 48, 11837−11845 (2014). 25216120 10.1021/es503306g

[b17] Bisaillon A., Beaudet R., Lepine F. & Villemur R. Quantitative analysis of the relative transcript levels of four chlorophenol reductive dehalogenase genes in *Desulfitobacterium hafniense* PCP-1 exposed to chlorophenols. Appl. Environ. Microb. 77, 6261−6264 (2011). 10.1128/AEM.00390-11 PMC3165378 21742910

[b18] Smits T. H. M., Devenoges C., Szynalski K., Maillard J. & Holliger C. Development of a real-time PCR method for quantification of the three genera *Dehalobacter, Dehalococcoides*, an *Desulfitobacterium* in microbial communities. J. Microbiol. Methods. 57, 369−378 (2004). 15134884 10.1016/j.mimet.2004.02.003

[b19] Loffler C. . Occurrence, genes and expression of the W/Se-containing class ΙΙ benzoyl-coenzyme a reductases in anaerobic bacteria. Environ. Microbiol. 13, 696−709 (2011). 21087381 10.1111/j.1462-2920.2010.02374.x

[b20] Kuntze K., Vogt C., Richnow H. H. & Boll M. Combined application of PCR-based functional assays for the detection of aromatic-compound-degrading anaerobes. Appl. Environ. Microb. 77, 5056−5061 (2011). 10.1128/AEM.00335-11 PMC3147403 21602396

[b21] Burbano C. S. . Predominant *nifH* transcript phylotypes related to *Rhizobium rosettiformans* in field-grown sugarcane plants and in Norway spruce. Environ. Microbiol. Rep. 3, 383−389 (2011). 23761284 10.1111/j.1758-2229.2010.00238.x

[b22] Mehta M. P., Butterfield D. A. & Baross J. A. Phylogenetic diversity of nitrogenase (*nifH*) genes in deep-sea and hydrothermal vent environments of the Juan de Fuca Ridge. Appl. Environ. Microb. 69, 960−970 (2003). 10.1128/AEM.69.2.960-970.2003 PMC143675 12571018

[b23] Ju X., Zhao L. & Sun B. Nitrogen fixation by reductively dechlorinating bacteria. Environ. Microbiol. 9, 1078−1083 (2007). 17359278 10.1111/j.1462-2920.2006.01199.x

[b24] Wu W. M., Bhatnagar L. & Zeikus J. G. Performance of anaerobic granules for degradation of pentachlorophenol. Appl. Environ. Microbiol. 59, 389−397 (1993). 8434908 10.1128/aem.59.2.389-397.1993 PMC202117

[b25] Li Z. L., Nan J., Yang J. Q., Jin X. & Katayama A. Temporal distributions of functional microbes and putative genes associated with halogenated phenol anaerobic dehalogenation and further mineralzaition. RSC Adv. 5, 89157–89163 (2015).

[b26] Chow W. L., Cheng D., Wang S. Q. & He J. Z. Identification and transcriptional analysis of trans-DCE-producing reductive dehalogenases in *Dehalococcoides* species. ISME J. 4, 1020–1030 (2010). 20357835 10.1038/ismej.2010.27

[b27] Desai M. S., Assig K. & Dattagupta S. Nitrogen fixation in distinct microbial niches within a chemoautotrophy-driven cave ecosystem. ISME J. 7, 2411−2423 (2013). 23924780 10.1038/ismej.2013.126 PMC3834856

[b28] Behrens S. . Monitoring abundance and expression of “*Dehalococcoides*” species chloroethene-reductive dehalogenases in a tetrachloroethene-dechlorinating flow column. Appl. Environ. Microbiol. 74, 5695−5703 (2008). 18676701 10.1128/AEM.00926-08 PMC2547056

[b29] Van De Pas B. A. . Purification and molecular characterization of *ortho*-chlorophenol reductive dehalogenase, a key enzyme of halorespiration in *Desulfitobacterium dehalogenans*. J. Biol. Chem. 274, 20287−20292 (1999). 10400648 10.1074/jbc.274.29.20287

[b30] Von Wintzingerode F., Schlotelburg C., Hauck R., Hegemann W. & Gobel U. B. Development of primers for amplifying genes encoding *cprA*- and *pceA*-like reductive dehalogenases in anaerobic microbial consortia, dechlorinating trichlorobenzene and 1,2-dichloropropane. FEMS Microbiol. Ecol. 35, 189−196 (2001). 11295458 10.1016/s0168-6496(01)00093-9

