# Supplementary material for: Spatial Abundance and Distribution of Potential Microbes and Functional Genes Associated with Anaerobic Mineralization of Pentachlorophenol in a Cylindrical Reactor
Source: Sci Rep. 2016 Jan 11;6:19015. doi: 10.1038/srep19015 (PMC4707460; doi:10.1038/srep19015)
Supplement: Supplementary Information [file srep19015-s1.doc]

**Supplementary information**

**Spatial Abundance and Distribution of Potential Microbes and Functional Genes Associated**

**with Anaerobic Mineralization of Pentachlorophenol in a Cylindrical Reactor**

**Authors:**

Zhiling Li^1,2^, Jun Nan^1^, Cong Huang^1^, Bin Liang^3^, Wenzong Liu^3^, Haoyi Cheng^3^, Chunfang Zhang^2^†, Dongdong Zhang^4^, Deyong Kong^1^, Kyoko Kanamaru^4^, Tetsuo Kobayashi^4^, Aijie Wang^1,3**^, and Arata Katayama^2,5*^

**Affiliation:**

^1^ State Key Laboratory of Urban Water Resources and Environment, Harbin Institute of Technology, Harbin 150090 China;

^2^ EcoTopia Science Institute, Nagoya University, Chikusa, Nagoya 464-8603 Japan;

^3^ Key Laboratory of Environmental Biotechnology, Research Center for Eco-Environmental Sciences, Chinese Academy of Sciences, Beijing 100085, P.R. China;

^4^ Department of Biological Mechanisms and Functions, Graduate School of Bioagricultural Sciences, Nagoya University, Chikusa, Nagoya 464-8601 Japan;

^5^ Department of Civil Engineering, Graduate School of Engineering, Nagoya University, Chikusa, Nagoya 464-8603 Japan.

† Present address: Ocean College, Zhejiang University, Hangzhou, Zhejiang, 310058, China

*Corresponding author:

Professor. Arata Katayama

E-mail: a-katayama@esi.nagoya-u.ac.jp

TEL: +81-52-789-5856 FAX: +81-52-789-5857

**Co-corresponding author:

Professor. Aijie Wang

E-mail: waj0578@hit.edu.cn

TEL: +86- 451-86282195

**Materials and Methods**

**Chemical analysis**

For PCP and the metabolites analysis, sample (1 mL of the total volume of soil and liquid) was acidified with 1N HCl, extracted with acetonitrile (1 mL) and ethyl acetate (2 mL), and then dehydrated with sodium sulfate anhydrous (Li et al., 2010). PCP and its aromatic metabolites were determined by GC-MS (Shimadzu, Kyoto, Japan) equipped with the capillary column (DB-5MS, 30m of length, 0.25 mm of inner diameter, J&W Scientific, Folsom, CA). For the analysis of organic acids, the sample (1 mL of the total volume of soil and liquid) was diluted by five times with distilled water, vibrated for 1 min to make the average distribution and filtered with 0.45 μM membrane filter before HPLC analysis. Organic acids (lactate, acetate and propionate) were determined using a high-performance liquid chromatograph (CTO-10A; Shimadzu, Kyoto, Japan) equipped with an ultraviolet detector (UV 280 nm) and a Puresil C18 column (4.6 mm inner diameter, 250 mm length; Waters, Milford, MA, USA), under a mobile phase of acetonitrile: H_2_O: acetic acid (65:34:1).

**DNA extraction and the primer design**

The *cprA* gene sequences applied for primers design included *cprA* (*Desulfitobacterium dehalogenans* ATCC 51507, *Desulfitobacterium* sp.Viet-1, *Desulfitobacterium hafniense* DCB-2, and *Dehalobacter restrictus* with accession numbers of AF115542, AAG49544, AAG46192, and CAC37166, respectively), *cprA1* (*Desulfitobacterium* sp. PCE1, *Desulfitobacterium chlororespirans* and *Desulfitobacterium hafniense* DCB-2 with accession numbers of AAG46187, AAL84925, AAG46192, and AF403182, respectively), *cprA2* (*Desulfitobacterium* sp. PCE1, *Desulfitobacterium chlororespirans* with accession numbers of AAG49543 and AAG43483 and *Desulfitobacterium hafniense* DCB-2 contig 3277), and *cprA3* (*Desulfitobacterium hafniense* DCB-2contig 3277, 3246 and *Desulfitobacterium hafniense* PCP-1 with accession numbers of YP_002457213).

**PCR, cloning and nucleotide sequencing analysis**

The PCR program for amplification of *cprA* genes, *nifH* genes, and benzoyl-CoA related genes were as follows: the initial denaturation step at 94°C for 2 min followed by 30 cycles of 94°C for 30s, 50°C for 1 min for primer annealing, and 72°C for 1 min for elongation, followed by a final elongation step at 72°C for 5 min. After amplification, the fragment with the desired size checked by agarose gel electrophoresis was separated by agarose gel electrophoresis, and purified with the high pure PCR products purification kit (Roche, Basel, Switzerland). Then products were cloned into *Escherichia coli* JM109 competent cells (Promega, Madison, WI, USA) with pGem-T easy cloning kit (Promega, Madison, WI, USA). The obtained transformants were screened by PCR amplification with primers SP6 (5′–ATTTAGGTGACACTATAGAATACTC–3′) and T7 (5′–TAATACGACTCACTATAGGGC–3′) and the ones with the correct sizes of insertions were digested with restriction endonuclease *Eco*RI (Takara Bio) and *Pst*I (Takara Bio) for 2 h at 37°C. The digested products were separated electrophoretically on 3% agarose gel and visualized with ethidium bromide under UV illumination. Sequences of the different types were analyzed with RDP classifier (<http://rdp.cme.msu.edu/classifier/classifier.jsp>) and compared with those in the GenBank nucleotide sequence database using the BLAST program (http://blast.ncbi.nlm.nih.gov/Blast.cgi). The phylogenetic tree with the different type of sequences and the closest related type strain was constructed with the software of MEGA 5.05 (http://www.megasoftware.net) using neighbor-joining algorithm incorporating Kimura 2-parameter distance correction.

**Quantitative PCR (*q*PCR) analysis**

Calibration curves (log DNA concentration versus an arbitrarily set cycle threshold value) for 16s rRNA genes and putative functional genes (*cprA*, *nifH* and *bamB* genes) were constructed using serial dilutions of amplicons of single colonies, obtained from setting up the 16S rRNA clone library with the bacterial consensus primers (27f and 1492r) from DNA in 0-2 cm of segment. Gene copy number of the amplicon was calculated by multiplying the molar concentration of the amplicon by Avogadro’s constant. The *q*PCR detection limits for Bacteria, Archaea, *Dehalobacter* sp., *Sulfurospirillum* sp., *Desulfitobacterium* sp., *Desulfovibrio* sp., *Cryptanaerobacter* sp., and *Syntrophus* sp. were 2.8 × 10^2^ copies mL^–1^, 1.9× 10^3^ copies mL^–1^, 1.1 × 10^3^ copies mL^–1^, 1.5 × 10^4^ copies mL^–1^, 1.5 × 10^3^ copies mL^–1^, 5.7× 10^2^ copies mL^–1^, 4.9× 10^3^ copies mL^–1^ and 2.9× 10^3^ copies mL^–1^, respectively. The *q*PCR detection limits for *cprA* genes, *nifH* genes and *bamB* genes were 2.5× 10^2^ copies mL^–1^, 2.6× 10^2^ copies mL^–1^, 3.7× 10^2^ copies mL^–1^, respectively.


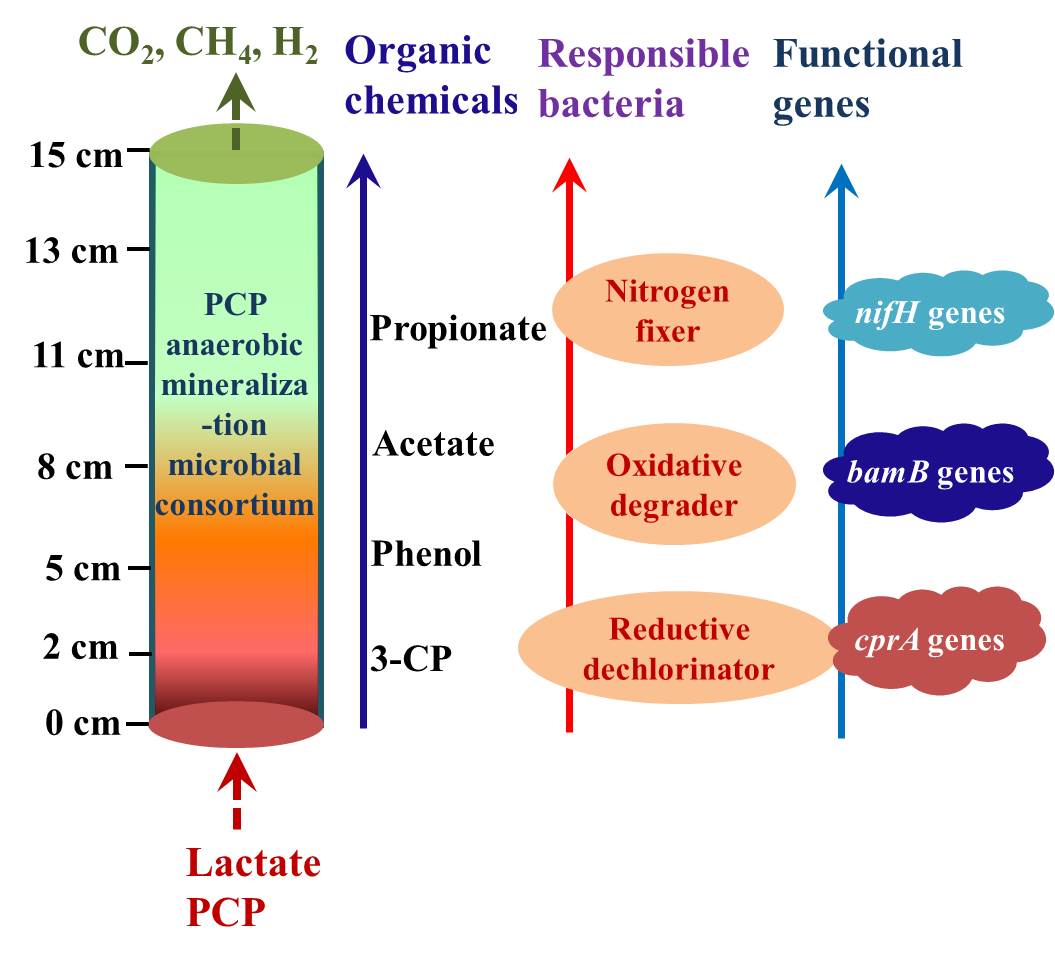


SI Figure S1: Framework of the reactor and the analytical details after reactor decomposition

SI Table 1. Oligonucleotide primers used for PCR reactions of *cprA*-like genes

| Name | Sequences (5’→3’) | Direction | Positions^*1^ | Gene targeted | References |
| --- | --- | --- | --- | --- | --- |
| f4-m3g1 | CRGAACYCTYGGYTAYAWTGC | Forward | 774-794 | CprAand CprA1 genes | This study |
| f4-m3g2 | CGCAATCTGGGYTAYMABGCC | Forward | 775-795 | CprA2,CprA3, and CprA4 genes | This study |
| f5-m3g1 | GGCTGCATTGCCGTCATTMTGGC | Forward | 742-764 | CprA and CprA1genes | This study |
| f5-m3g2 | TATCATGCYACAGCYRWYATTTCC | Forward | 730-753 | CprA2,CprA3, and CprA4 genes | This study |
| r3-m3g1 | CCATAVCCRAAGATATCATC | Reverse | 1255-1274 | CprAand CprA1 genes | This study |
| r3-m3g2 | CCRTAGCCRAACATATCGTC | Reverse | 1255-1274 | CprA2,CprA3, and CprA4 genes | This study |
| r4-m1 | BGCYTYATGGAACCAGG | Reverse | 1184-1200 | CprA genes | This study |
| CprA3f | AAGAAATGCGCCGAATTCCGTCTG | Forward |  | CprA3(*D. hafniense* PCP-1) | Bisaillon et al., 2011 |
| CprA3r | ATCCAAATGCCGGCTGAATGGAAC | Reverse |  | CprA3(*D. hafniense* PCP-1) | Bisaillon et al., 2011 |
| CprA4f | TGACCAATGGCTTGGTACCTCGAA | Forward |  | CprA4(*D. hafniense* PCP-1) | Bisaillon et al., 2011 |
| CprA4r | TAGCCATTCCCATGGCATCACTCA | Reverse |  | CprA4(*D. hafniense* PCP-1) | Bisaillon et al., 2011 |
| CprA5f | TTAAAGCATCTCTGGTGAGCGTCG | Forward |  | CprA5(*D. hafniense* PCP-1) | Bisaillon et al., 2011 |
| CprA5r | ACTTGGTAAGGAAATTCCGCGGTC | Reverse |  | CprA5(*D. hafniense* PCP-1) | Bisaillon et al., 2011 |

^*1^ Numbers refers to the *cprA* gene of *Desulfitobacterium dehalogenans* ATCC 51507 (Van De Pas et al., 1999).

SI Table 2. Oligonucleotide primers used for PCR reactions of nitrogenase gene, benzoyl-CoA reductase genes and

ring-cleaving hydrolasegenes of benzoyl-CoA.

| Name | Sequences (5’→3’) | Direction | Length (bp) | Gene targeted | References |
| --- | --- | --- | --- | --- | --- |
| NifH-f | GGHAARGGHGGHATHGGNAARTC | Forward | 400 | All *nifH*, *anfH*, *vnfH*, and archaeal　*nifH*^*1^ | Mehta et al., 2003 |
| NifH-r | GGCATNGCRAANCCVCCRCANAC | Reverse |  |  |  |
| BamBf | ATGMGGTAYGSAGARACHGG | Forward | 320 | Benzoyl-coenzyme A reductases in obligate anaerobes ^*2^ | Loffler et al., 2011 |
| BamBr | CCSGCRWRYTTCADYTCCG | Reverse |  |  |  |
| BzdNf | GAGCCGCACATCTTCGGCAT | Reverse | 700 | *Thauera*type of benzoyl-CoA reductasein facultative anaerobes | Kuntze et al., 2011 |
| BzdNr | TRTGVRCCGGRTARTCCTTSGTCGG | Reverse |  |  |  |
| BcrCf | BGCYTYATGGAACCAGG | Reverse | 800 | *Azoarcus* type of benzoyl-CoA reductasein facultative anaerobes | Kuntze et al., 2011 |
| BcrCr | CGGATCGGCTGCATCTGGCC | Forward |  |  |  |
| BamA-SP9 | CAGTACAAYTCCTACACVACBG | Reverse |  | Ring-cleaving hydrolase of the benzoyl-CoA | Abu laban., 2010 |
| BamA-ASP1 | CMATGCCGATYTCCTGRC | Forward | 300 |  |  |
| BamA-ASP23 | TTTTCCTTGTTGVSRTTCC | Reverse | 700 |  | Kuntze et al., 2011 |
| BamA-ASP33 | CAKYYSGGGAASAGRTTKG | Reverse | 800 |  | Kuntze et al., 2011 |

^*1^:The *nifH* encodes the iron protein in nitrogenase. The one contained alternative nitrogenases in which the dinitrogenase protein contains vanadium instead of molybdenum, encoded by *vnf* genes, or only iron, encoded by the *anf* genes.

^*2^: The *bamB* belongs to the aldehyde: ferredox inoxidoreductase (AOR) family and contains an active site tungstopterin and a [4Fe-4S] cluster.

SI Table 3. Oligonucleotide primers for *q*PCR analysis

|  | Sequences (5' →3') | Position | Product size (bp) | Target organics | Reference or source |
| --- | --- | --- | --- | --- | --- |
| Eub341F | CCTACGGGAGGCAGCAG | 341-357 | 194 | Bacteria | Muyzer et al., 1993 |
| Eub534R | ATTACCGCGGCTGCTGG | 534-517 |
| Ar344F | ACGGGGTGCAGGCGCGA | 344-360 | 191 | Archaea | Muyzer et al., 1993 |
| Ar534R | ATTACCGCGGCTGCTGG | 534-517 |
| Dre441F | GTTAGGGAAGAACGGCATCTGT | 441-461 | 227 | *Dehalobacter* spp. | Smits et al., 2004 |
| Dre645R | CCTCTCCTGTCCTCAAGCCATA | 666-645 |
| Dsb406F | GTACGACGAAGGCCTTCGGGT | 406-426 | 215 | *Desulfitobacterium* spp. | Smits et al., 2004 |
| Dsb619R | CCCAGGGTTGAGCCCTAGGT | 619-600 |
| Sulfuro114F | GCTAACCTGCCCTTTAGTGG | 114-137 | 308 | *Sulfurospirillum* spp. | Duhamel and Edwards, 2006 |
| Sulfuro421R | GTTTACACACCGAAATGCGT | 421-402 |
| Dsfv1131F | GCGAGTAATGTCGGGCACTCTAG | 1131-1152 | 181 | *Desulfovibrio* spp. | This study |
| Dsfv1310R | CCGATCCGGACTGGGATG | 1310-1293 |
| Cry584F | GGCGGTCATTTAAGTCAGAGG | 584-604 | 292 | *Cryptanaerobacter* spp. | This study |
| Cry875R | CTTATTGCGTTAGCTACGGC | 875-856 |
| Syn195F | AAAGAGAGCCTCTGCWTGCAAGCCC | 195-215 | 303 | *Syntrophus* spp. | This study |
| Syn497R | CCTTTGGCGGTACCGTCAAGTAC | 497-475 |

SI Table 4. Primer pairs showing the amplified products of *cprA-like* genes from the DNA sample in 0-2 cm

| Primer pair | Length (bp) | Amplification | Primer pair | Length (bp) | Amplification |
| --- | --- | --- | --- | --- | --- |
| f4-m3-g1/r3-m3-g1 | 500 | +^*1^ | f5-m3-g1/r4-m1 | 459 | +^*3^ |
| f4-m3-g2/r3-m3-g2 | 501 | +^*2^ | f5-m3-g2/r4-m1 | 471 | +^*4^ |
| f5-m3-g11/r3-m3-g1 | 533 | + | CprA3f/CprA3r | 119 | – |
| f5-m3-g2/r3-m3-g2 | 545 | – | CprA4f/CprA4r | 861 | – |
| f4-m3-g1/r4-m1 | 427 | + | CprA5f/CprA5r | 139 | – |
| f4-m3-g2/r4-m1 | 426 | + |  |  |  |

^*1^:Amplicon was used to construct clone library PCP-1;^*2^:Amplicon was used to construct clone library PCP-2;^*3^:Amplicon was used to construct clone library PCP-3;^*4^:Ampliconwas used to construct clone library PCP-4.

SI Table 5. Sequence identity of putative *cprA* genes obtained from clone library analysis in the continuous-flow cylindrical bioreactor

| Clones | Sequence Identity (%) | | | | |
| --- | --- | --- | --- | --- | --- |
|  | *Desulfitobacteriumchlororespirans,o*-chlorophenol CprA (AF204275) | *D.dehalogenans*,  *o*-chloro/bromophenol  CprA (AF115542) | *D.hafniense* PCP-1, CprA3 (CP001336) | *D.hafniense* PCP-1 *p*- and *m*-chlorophenol CprA5 (AY349165) | *Dehalobacter restrictus*, CprA-like (CAC37166) |
| Group1-f4r3g1-1 | 75.7 | 70.1 | 68 | - | 71.9 |
| Group2-f4r3g2-1 | 71.1 | 75 | 74.9 | - | 74.7 |
| Group3-f4r3g1-3 | 67.7 | 73.2 | 71.1 | - | 70.9 |
| Group3-f5r4g1-2 | 70.3 | 74.2 | 72.1 | - | 71.1 |
| Group4-f4r3g1-4 | 70.1 | 72.8 | 69.6 | - | 73.8 |

SI Table 6. PCR-amplificationresults of phenol anaerobic oxidase genes and nitrogenase genes

| Primer pair | Amplification with DNA in inlet section (0-2 cm) | Amplification with DNA in outlet section (13-15 cm) |
| --- | --- | --- |
| BamBf-BamBr | +^*1^ | – |
| BzdNf-BzdNr | – | – |
| BcrCf-BcrCr | – | – |
| BamA-SP9-ASP1 | – | – |
| BamA-SP9-ASP1 | – | – |
| BamA-SP9-ASP1 | – | – |
| NifH-f-NifH-r | +^*2^ | +^*3^ |

^*1^: Amplification was used to construct clone library BamB; ^*2^: Amplification was used to construct clone library NifI;^*3^: Amplification was used to construct clone library NifO.

**Reference**

Abu Laban, N., Selesi, D., Rattei, T., Tischler, P. & Meckenstock, R. U. Identification of enzymes involved in anaerobic benzene degradation by a strictly anaerobic iron-reducing enrichment culture. *Environ. Microbiol.* **12**, 2783–2796 (2010).

Bisaillon, A., Beaudet, R., Lepine, F. & Villemur, R. Quantitative analysis of the relative transcript levels of four chlorophenol reductive dehalogenase genes in *Desulfitobacterium hafniense* PCP-1 exposed to chlorophenols. *Appl. Environ. Microb.* **77**, 6261−6264 (2011).

Mehta, M.P., Butterfield, D.A. & Baross, J.A. Phylogenetic diversity of nitrogenase (*nifH*) genes in deep-sea and hydrothermal vent environments of the Juan de Fuca Ridge. *Appl. Environ. Microb.* **69**, 960−970 (2003).

Loffler, C., Kuntze, K., Vazquez, J.R., Rugor, A., Kung, J.W., Bottcher, A. & Boll, M. Occurrence, genes and expression of the W/Se-containing class ΙΙ benzoyl-coenzyme a reductases in anaerobic bacteria. *Environ. Microbiol.* **13**, 696−709 (2011).

Kuntze, K., Vogt, C., Richnow, H.H. & Boll, M. Combined application of PCR-based functional assays for the detection of aromatic-compound-degrading anaerobes. *Appl. Environ. Microb.* **77**, 5056−5061 (2011).

Muyzer, G., DeWaal., E.C. & Uitterlinden, A.G. Profiling of complex microbial populations by denaturing gradient gel electrophoresis analysis of polymerase chain reaction-amplified genes coding for 16S rRNA. *Appl. Environ. Microbiol.* **59**, 695−700 (1993).

Smits, Theo H.M., Devenoges, C., Szynalski, K., Maillard, J. & Holliger, C. Development of a real-time PCR method for quantification of the three genera *Dehalobacter*, *Dehalococcoides*, and *Desulfitobacterium* in microbial communities. *J Microbiol. Meth.* 57, 369−378 (2004).

Duhamel, M. & Edwards, E.A. Microbial composition of chlorinated ethene-degrading cultures

dominated by *Dehalococcoides*. *FEMS Microbiol. Ecol.* 58, 538–549 (2006).
